# Editorial: eSports and digitalization of sports

**DOI:** 10.3389/fspor.2022.1040468

**Published:** 2022-09-23

**Authors:** Ivo van Hilvoorde

**Affiliations:** ^1^MOVE Research Institute Amsterdam, VU Medical Center, Amsterdam, Netherlands; ^2^Research Centre for Exercise, School and Sport, Windesheim University of Applied Sciences, Zwolle, Netherlands

**Keywords:** eSport, digitalization, technologies, integrity, values, governance, Olympic games, IOC

The increasing development of eSports and digitalization in traditional sports are major features among the various changes which occurred in the field of sports over the last two decades. First, the ever-growing eSports industry contributes to redefine the boundaries of sports and physical activity, and eSports today aims at being eligible for inclusion in the Olympic programme and other major international sports events. eSports competitions also increasingly attract fans in packed arenas and/or on streaming platforms, then providing new avenues for business expansion of the sports sector. Such opportunities also include brand extension strategies resulting in the development of eSports sections within well-established sports clubs (e.g., football clubs). Meanwhile, the development of eSports is raising several sports-integrity related issues, such as fairness, doping, match-fixing, discrimination, and violence. It also raises gender-related questions and challenges related to the social norms surrounding the development of gamers' communities.

Not only does the existence of eSports across digital spaces makes the challenge of digitalization of sports very topical. In fact, the digitalization process of sports plays an important role in helping transform the outline of both elite and grassroot sports. The increasing use of measurement and monitoring tools of athletic performance (e.g., power meters, connected watches, smart home trainers) tends to modify how athletic performance is produced, as well as how athletes interact with both their support personnel and followers through technology and metrology. This process of digitalization of sports may also have an impact on athlete's involvement in the practice and (perceived) autonomy since it shapes body surveillance under continuous monitoring. The various effects of the increasing use of technologies and metrologies in sports may then be addressed by the academic scholarship.

The objective of this Research Topic is to gather updated scientific and multidisciplinary contributions addressing the challenges and issues related to the increasing development of eSports, and the effects of the digitalization process in sports. The contributions of this Research Topic include a variety of research areas, ranging from sports sociology to sport ethics and management. The four papers of this issue deal with a variety of topics, including debates on definition, embodiment, recognition and institutionalization of eSports, governance of eSports, digital consumer behavior in sports, changes in organizational cultures through digitalization, spectatorship, fandom, integrity and regulation of eSports.

Heidenreich et al. discuss some of the fundamental differences between the governance of eSports and traditional sports. Traditional sports are usually non-profit associations, with the core task of developing and safeguarding the sport by regulations, rules and licensing. eSports are governed by profit corporations. Publishers develop and market the electronic games as commercial products with exclusive property rights. This means that publishers control the rules of the virtual sporting environment. This paper is based upon an empirical study (content analysis of 55 documents and nine interviews with relevant stakeholders) of the emergence and legitimacy of two eSports associations (the World eSports Association and the eSport-Bund Deutschland). Key findings of this study are that eSports associations are struggling for recognition and acceptance and need to accept the dominance of the publishers.

In his article Ekdahl deals with the claim that eSports fails to be sports because it is supposed to be not “direct” or “immediate,” compared to so-called “physical sports.” The author further elaborates on the relationship between eSports and physicality, and connects this with the question what kind of motor skills are learned in eSports. This paper offers an alternative way of thinking about body and space. Focussing on the subjective, bodily engagement of eSports practitioners, the physical and virtual space is experienced and appreciated as immediate, and directly interconnected with the (“virtual”) performance.

Postma et al. deal with the question which eSports can or should be included in the Olympic Program. In 2021, the IOC already launched the Olympic Virtual Series, featuring virtual and wellknown sports such as baseball, cycling, rowing, sailing and motor racing. This begs the question why popular eSports like Fortnite, League of Legends or Dota are excluded from this series. The authors argue that the question of inclusion within the Olympics should not be based upon the question if it looks like “real sport” as we know it, or upon the false distinction between “physical” and “non-physical,” but rather upon a discussion of Olympic values, such as the values of meritocracy, competition, fair play, and having a “level playing field.” These values should ideally be discussed in relation to issues of designing the games, whereby *Value Sensitive Design* can be a valuable tool.

Huettermann and Pizzo are interested in eSports fans and studied differences in eSports fan engagement. Differences in games, genres and platforms influences the behavior and consumption of eSports players. Why do fans spectate, support or follow eSports teams and players? In their paper, the authors identify relevant differences that influence eSports consumer behavior. Based upon data from eSports team fans (*via* an online survey) the authors identify differences in how fans of PC and console based eSports teams engage with their favorite eSports team. The results illustrate that eSports fans and teams value both emotional engagement, more intimate experiences and cooperation. This implies that the platform (personal computers, video gaming consoles) plays an important role in the engagement of eSports teams and players.

## Author contributions

The author confirms being the sole contributor of this work and has approved it for publication.

## Conflict of interest

The author declares that the research was conducted in the absence of any commercial or financial relationships that could be construed as a potential conflict of interest.

## Publisher's note

All claims expressed in this article are solely those of the authors and do not necessarily represent those of their affiliated organizations, or those of the publisher, the editors and the reviewers. Any product that may be evaluated in this article, or claim that may be made by its manufacturer, is not guaranteed or endorsed by the publisher.

